# Exploring the relationship between white matter integrity, cocaine use and GAD polymorphisms using Bayesian Model Averaging

**DOI:** 10.1371/journal.pone.0254776

**Published:** 2021-07-26

**Authors:** Tmader Alballa, Edward L. Boone, Liangsuo Ma, Andrew Snyder, F. Gerard Moeller

**Affiliations:** 1 Department of Statistical Sciences and Operations Research, Virginia Commonwealth University, Richmond, Virginia, United States of America; 2 Department of Psychiatry, Virginia Commonwealth University, Richmond, Virginia, United States of America; 3 Institute of Drug and Alcohol Studies, Virginia Commonwealth University, Richmond, Virginia, United States of America; 4 Mathematical Sciences Department, College of Science, Princess Nourah bint Abdulrahman University, Riyadh, Saudi Arabia; 5 Department of Pharmacology and Toxicology, Virginia Commonwealth University, Richmond, Virginia, United States of America; 6 Department of Neurology, Virginia Commonwealth University, Richmond, Virginia, United States of America; 7 C. Kenneth and Dianne Wright, Center for Clinical Translational Research, Virginia Commonwealth University, Richmond, Virginia, United States of America; McLean Hospital, UNITED STATES

## Abstract

Past investigations utilizing diffusion tensor imaging (DTI) have demonstrated that cocaine use disorder (CUD) yields white matter changes, primarily in the corpus callosum. By applying Bayesian model averaging using multiple linear regression in DTI, we demonstrate there may exist relationships between the impaired white matter and glutamic acid decarboxylase (*GAD*) polymorphisms. This work explored the two-way and three-way interactions between *GAD*1^*a*^ (SNP: rs1978340) and *GAD*1^*b*^ (SNP: rs769390) polymorphisms and years of cocaine use (*YCU*). *GAD*1^*a*^ was associated with more frontal white matter changes on its own but *GAD*1^*b*^ was associated with more midbrain and cerebellar changes as well as a greater increase in white matter changes in the context of chronic cocaine use. The three-way interaction *GAD*1^*a*^|*GAD*1^*b*^|*YCU* appeared to be roughly an average of the polymorphism two-way interactions *GAD*1^*a*^|*YCU* and *GAD*1^*b*^|*YCU*. The three-way interaction demonstrated multiple regions including corpus callosum which featured fewer significant voxel changes, perhaps suggesting a small protective effect of having both polymorphisms on corpus callosum and cerebellar peduncle.

## Introduction

Cocaine use disorder (CUD) patients exhibit altered white matter integrity versus non-drug using controls [1–7], with the most consistent findings in the corpus callosum. The altered white matter integrity is particularly evident in CUD patients with greater severity of cocaine use [4]. Moreover, the changes in the white matter have also been associated with the length of cocaine use [8]. Diffusion tensor imaging (DTI) is a non-invasive magnetic resonance imaging (MRI) technology suitable for characterizing microstructural changes in human neuropathology; DTI measures restricted diffusion of water due to cellular membranes and organelles that comprise the structural integrity of brain [9]. Fractional anisotropy (FA), the most commonly used DTI measure, describes the macroscopic axonal organization in brain with lower FA generally suggesting reduced white matter integrity [1, 10]. Consistent with these observations, preclinical studies have shown that lower FA is observed in rats exposed to a chronic regimen of cocaine [11, 12], supporting the hypothesis that exposure to cocaine may underlie the lower FA seen in human CUD patients. However, not all CUD patients show reduced FA compared to healthy controls (e.g., [13]), and few studies to date have examined factors that could enhance or reduce the impact of cocaine on white matter integrity.

However, a previous study [14] tested twenty-one genetic variants across seventeen candidate genes that may be related to cocaine addiction, where Bayesian Model Averaging with linear regression was used to explore the effects of the cocaine use on these genetic variants. Their DTI findings were related to two Single Nucleotide Polymorphisms (SNPs) *GAD*1^*a*^ and *GAD*1^*b*^. That previous study [14] finds that comparing the impact of *GAD*1^*a*^ to the other 20 genetic variants on diffusion in the white matter of the brain was comprehensive with approximately 4 times more in the number of significant voxels compared to the second most effective genetic variant. However, that study only looked at single genes without looking at any interactions between SNP’s and years of cocaine use in the model. The purpose of this study is to further explore how the *GAD*1^*a*^ and *GAD*1^*b*^ SNPs in human brain cells interact with human sex, years of cocaine use, and white matter changes as measured by FA in DTI using a Bayesian Model Averaging [15] whit linear regression model [16] by using exactly the same data-set that have been used in the previous work [14]. BMA is one of the most effective strategies that deals with model uncertainty, which takes into account all possible models. The novel contribution of this study is the demonstration of a series of two-way *GAD*1^*a*^|*YCU* / *GAD*1^*b*^|*YCU* and three-way *GAD*1^*a*^|*GAD*1^*b*^|*YCU* models which enable us to assess the individual contributions of each covariate in determining white matter changes in CUD.

## Study population

Informed consent was obtained from each patients and healthy controls before being included in this study. Treatment-seeking CUD patients and healthy controls were recruited via newspaper advertisements and were initially screened by a brief telephone interview. Following the phone screen, eligible individuals attended an in-person intake assessment session, during which screening for psychiatric disorders using the Structured Clinical Interview for DSM-IV [17], and a medical history and physical examination were conducted. Information about each individual’s (drug and non-drug user) demographic and drug use history was also collected at the intake interview. For all individuals, the Addiction Severity Index [18] was obtained to document lifetime drug and alcohol use. Immediately prior to MRI scanning, a urine sample was obtained from each individual to screen for Δ^9^-tetrohydrocannabinol, opiates, cocaine, amphetamines, benzodiazepines, and pregnancy (for females). Each individual was also screened for recent alcohol use by measuring breath alcohol concentration. Individual inclusion criteria were:

1. 18 to 55 years old.
2. free of alcohol at the time of MRI scanning.
3. CUD patients met criteria for current cocaine dependence as determined by Structured Clinical Interview for DSM-IV (SCID) [17].
4. healthy controls had no current or lifetime history of any DSM-IV substance use or psychiatric disorder.

Exclusion criteria were:

1. met current or past DSM-IV Axis I disorder other than substance abuse or dependence.
2. taking medication or having disorders that could affect the central nervous system.
3. claustrophobia during MRI simulator sessions.
4. having any definite or suspected clinically-significant brain abnormalities on the Fluid-Attenuated Inversion Recovery (FLAIR) MRI scans.
5. urine drug screen showing positive for Δ^9^-tetrohydrocannabinol, opiates, cocaine, amphetamines, benzodiazepines (for controls only).
6. positive pregnancy test result (for females).

Based on the inclusion and exclusion criteria, 39 CUD patients and 18 healthy controls were included in the final analysis. Demographic, drug use, and genotype characteristics of the two groups are shown in Table 1.

**Table 1:** Range and average for demographics of study sample in all groups.

Group	Sex	N	Age	YCU	Average of years	*GAD*1^*a*^	*GAD*1^*b*^
Cocaine use disorder	M	29	(23.1–52.5)	(0.25–30)	14.112	0.655	0.862
	F	10	(22.7–54.8)	(2–23)	13.00	0.5	0.8
	All	39	(22.7–54.8)	(0.25–30)	13.8269	0.615	0.846
Control	M	11	(23.2–48.8)	NA	NA	0.417	0.667
	F	7	(21–42.1)	NA	NA	0.428	1
	All	18	(21–48.8)	NA	NA	0.421	0.789

N: sample size, Age and Years of use Cocaine: range in years, YCU: Years of cocaine use.

## MRI data acquisition

MRI data were acquired on a Philips 3.0 T Intera system with a six channel receive head coil (Philips Medical Systems, Best, Netherlands). Whole brain diffusion-weighted images (DWI) were acquired in the transverse plane using a single shot diffusion sensitized spin echo echo-planar imaging (EPI) sequence, with the following parameters: b-factor = 1000 *s*/*mm*^2^, repetition time = 6100 *ms*, echo time = 84 *ms*, 44 contiguous axial slices, field-of-view = 240 *mm* × 240 *mm*, 112 × 112 acquisition matrix, 256 × 256 reconstructed matrix, 0.9375 *mm* × 0.9375 *mm* reconstructed in-plane resolution, slice thickness = 3 *mm*, and zero interslice gap. The diffusion tensor encoding scheme was based on the uniformly distributed and balanced rotationally invariant Icosa21 (21 gradient directions) tensor-encoding set [19]. A SENSE acceleration factor of 2 was used for the DWI acquistion. The diffusion-encoded volumes were acquired with fat suppression. The DTI acquisition time was approximately 7 min. FLAIR scans and T2-weighted spin-echo scans were acquired and were read by a board-certified radiologist to rule-out any incidental brain pathology.

## DTI data processing

The DTI images were processed using the FMRIB Software Library www.fmrib.ox.ac.uk/fsl,version5.0.9 [20]. For each data set, the DWI images were corrected for eddy current distortions and head motion [21] after converting the Philips DICOM files into NIfTI format using dcm2nii as implemented in MRIcron http://www.mccauslandcenter.sc.edu/mricro/mricron/. Next, non-brain tissue was removed from the images using FSL’s Brain Extraction Tool [22]. After these preprocessing steps, the FMRIB’s Diffusion Toolbox [23] was used to fit the data to extract the DTI parameters for each voxel. The DTI parameters included fractional anisotropy (FA), mean diffusivity (MD), the three eigenvalues (*L*1, *L*2, *L*3) and the three eigenvectors (*V*1, *V*2, *V*3). Among these DTI measures, FA has been most commonly used as a general measure of white matter microstucture [24].

White matter registration was carried out using Tract-Based Spatial Statistics (TBSS) [25] as implemented in the FSL software. The FA images from all individuals were aligned to the standard MNI (Montreal Neurological Institute) space using the FSL’s nonlinear registration with the FMRIB58_FA template image. Next, the mean FA image was created and thinned to create a mean FA skeleton representing the centers of all tracts common to all individuals, using a threshold of FA = 0.20. Each individual’s aligned FA data that were local maximums within a short-radius plane normal to each point on the skeleton which were then projected onto that point of the skeleton.

## Genetic data acquisition

DNA was extracted from the blood samples drawn from each subject using standard methods [26]. The candidate genetic variants *GAD*1^*a*^ (SNP: rs1978340) and *GAD*1^*b*^ (SNP: rs769390) were determined as described in previous studies [14, 27].

## Method

### Model formulation

This study was approved by the local university Committee for the Protection of Human Participants (CPHS) and was performed in accordance with the Code of Ethics of the World Medical Association (Declaration of Helsinki). This is a secondary data analysis, the data is the same data from a previous work [14]. This work is performed on a data-set of 57 individuals of which 39 were cocaine dependent and 18 non-drug using controls. In this study, the covariates that are considered in the model are *GAD*1^*a*^, *GAD*1^*b*^, human sex, and years of cocaine use, and their interactions. That leaves us with eleven covariates (*k* = 11). The following set of covariates were considered in the regression model:

$$\begin{array}{c} \{\;Sex,Y\;CU,GAD1^{a},GAD1^{b},\\ Sex|Y\;CU,Sex|GAD1^{a},Sex|GAD1^{b},\\ Y\;CU|GAD1^{a},Y\;CU|GAD1^{b},GAD1^{a}|GAD1^{b},\\ Y\;CU|GAD1^{a}|GAD1^{b}\;\} \end{array}$$

Let the response variable *y*_*i*_(*v*) be the FA values at specific voxel *v* = 1, …, *V* for *i* = 1, …, *n* where *n* is the number of brain images (patients), and let *X* be the regression *n* × *k* matrix with demographic and genetic variables, knowing that covariates do not vary between voxels. So, the general linear regression model for the *V*^*th*^ voxel is given by *y*(*v*) = *Xβ*(*v*) + *ϵ*(*v*), where *ϵ* ∼ *N*(0, *σ*^2^(*v*)), and *β*(*v*) is the vector of regression coefficients.

### Bayesian model averaging

The model space is **M** = {*M*_*j*_: *j* = 1, …, 2^*k*^}, where 2^*k*^ = 2^11^ = 2048 is the number of all possible model combinations, and any *M*_*j*_ model has a subset of demographic and genetic variables. Since the covariates are the same among all voxels, the model space does not vary through all voxels. Let *M*_*j*_ be any model in **M** with parameters $\theta_{j}\left(v\right)=\left(\beta_{j}\left(v\right),\sigma_{j}^{2}\left(v\right)\right)$ at voxel *v*. Where *β*_*j*_(*v*) is the vector of regression coefficients and $\sigma_{j}^{2}\left(v\right)$ is the variance for the *j*^*th*^ model. The prior probability for any *j*^*th*^ model in the model space **M** indicate as *p*(*M*_*j*_). Since we do not have any prior knowledge and we do not prefer any model on others, so we assume $p\left(M_{j}\right)=\frac{1}{\left|M\right|}=\frac{1}{2048}$ as uniform distribution among the model space with size |**M**| = 2048. To obtain a posterior probability for model *M*_*j*_ at voxel *v* under data *D*(*v*) = (*Y*(*v*), *X*), we use Bayes theorem:

$$\begin{array}{c} p\left(M_{j}|D\left(v\right)\right)=\frac{p\left(D\left(v\right)|M_{j}\right)p\left(M_{j}\right)}{\sum_{j=1}^{2^{K}}p\left(D\left(v\right)|M_{i}\right)p\left(M_{i}\right)} \end{array}$$ (1)

where *p*(*D*(*v*)|*M*_*j*_) is the marginal probability of the data given model *M*_*j*_:

$$\begin{array}{c} p\left(D\left(v\right)|M_{j}\right)=\int L\left(D\left(v\right)|\theta_{j},M_{j}\right)p\left(\theta_{j}|M_{j}\right)d\theta_{j} \end{array}$$ (2)

Here *θ*_*j*_ is the group parameters that associated with model *M*_*j*_, and *p*(*θ*_*j*_|*M*_*j*_) is the prior distribution of *θ*_*j*_ for model *M*_*j*_. For the parameters that common to all models corresponding to a given voxel such as the intercept *β*_0*j*_ and the variance $\sigma_{j}^{2}$, we used a statndard non-informative prior $p\left(\beta_{0j},\sigma_{j}^{2}\right)=\sigma_{j}^{-1}$ [28]. In the special case when the prior for the regression coefficients is $\beta_{j}\left(v\right)∼N\left(0,\sigma^{2}{\left(gX_{j}^{\prime }X_{j}\right)}^{-1}\right)$, where $g=\frac{1}{max\{n,k^{2}\}}$, then according to [29] there exists a closed form for *p*(*D*(*v*)|*M*_*j*_) as:

$$\begin{array}{c} \begin{array}{cc} p(D\left(v\right)|M_{j}) & ∼{\left(\frac{g}{g+1}\right)}^{\frac{k_{j}}{2}}(\frac{1}{g+1}y{\left(v\right)}^{\prime }\left[I_{n}-X_{j}{\left(X_{j}^{\prime }X_{j}\right)}^{-1}X_{j}^{\prime }\right]y\left(v\right)\\ & +\frac{g}{g+1}{\left(y\left(v\right)-\bar{y(v)}\right)}^{\prime }{\left(y\left(v\right)-\bar{y(v)}\right)}^{\frac{-(n-1)}{2}} \end{array} \end{array}$$ (3)

where $\bar{y(v)}$ is a vector of length n with the sample mean of *y*(*v*) for each voxel, and *k*_*j*_ is the number of covariates in the *j*^*th*^ model. In BMA, measuring variables importance depends on the posterior effect probability of each variable. After calculating the posterior probabilities for all models, we compute the Posterior Inclusion Probability (PIP) for every covariate as well by:

$$\begin{array}{c} \pi_{k}\left(v\right)=p\left(X_{k}|D\left(v\right)\right)=\sum_{i=1}^{2^{k}}I_{X_{k}\in M_{j}}\left(M_{j}\right)p\left(M_{j}|D\left(v\right)\right) \end{array}$$ (4)

Where *p*(*M*_*j*_|*D*(*v*)) is the posterior model probability for *M*_*j*_, and $I_{X_{j}\in M_{j}}\left(M_{j}\right)$ is a function that takes only two values 0 and 1. It takes value 1 if *X*_*j*_ ∈ *M*_*j*_ and 0 otherwise [30]. Finally, we compute the posterior distribution of coefficients *β*_*j*_(*v*) to determine the sign of effect at the brain locations that have high PIP by:

$$\begin{array}{c} p\left(\beta_{j}\left(v\right)|D\left(v\right)\right)=\sum_{j=1}^{2^{k}}p\left(\beta_{j}\left(v\right)|D\left(v\right),M_{j}\right)p\left(M_{j}|D\left(v\right)\right) \end{array}$$ (5)

However, calculating the posterior probabilities for any model *M*_*j*_ is difficult to implement since it depends on the number of models which is in our case 2048 models for each voxel, that is involved in the summation to calculate the posterior probability. The Markov chain Monte Carlo model composition procedure (*MC*^3^) can evaluate the posterior model probabilities. According to [31] this approach randomly moves through the model space **M**, assuming that we start at model *M*_*j*_ with a neighborhood named $N_{M_{j}}$. Where $N_{M_{j}}$ contains the model *M*_*j*_ itself and a group of other models with either one variable more or one variable fewer than *M*_*j*_. Let $M_{m}\in N_{M_{j}}$ a new model, which chosen randomly by sampling from a uniform distribution on the $N_{M_{j}}$. Suppose that the chain starts at model *M*_*j*_, the chain might move to *M*_*m*_ with probability

$$\begin{array}{c} \omega_{jm}=min\left\{1,\frac{p(M_{m}|y\left(v\right))}{p(M_{j}|y\left(v\right))}\right\} \end{array}$$ (6)

Otherwise, the chain stays at model *M*_*j*_ with probability 1 − *ω*_*jm*_. The procedure continues by randomly finding a new model along the existing model. In the model selection, the model *M*_*j*_ with the highest values of *p*(*M*_*j*_|*y*(*v*)) is selected to be the best model. For more details describing the Markov chain Monte Carlo see [32].

### Bayesian false discovery rate control

The aforementioned method used to find brain regions that present strong evidence for differential diffusion patterns across the specific genetic and demographic variables. There are over 600, 000 voxels in this work,that number of voxels consider as high number and would cause a high false discovery rate. By apply the scheme developed by [33, 34], the False Discovery Rate (FDR) issue is resolved. Now assume that the remain voxels determine specific locations where the corresponding FA are affected by the differences in a particular genetic variant where the posterior probabilities exceed a given threshold *ϕ*. First need to find these posterior probabilities *π*_*k*_(*v*), suppose the space of those significant voxels for one specific genetic variant is given by:

$$\begin{array}{c} X_{\phi }=\left\{v:\pi_{k}\left(v\right)\geq \phi \right\} \end{array}$$ (7)

Let *ϕ*_*δ*_ is the corresponding threshold where *δ* represent the chosen level for the false discovery rate, which in our case is 10% of the highest significant voxels. To determine which probability needs to include at that space want to choose *ϕ*. Suppose Ψ is the space of all our posterior probabilities, and then sorting Ψ descending. Our threshold *ϕ*_*δ*_ = Ψ_*k*_(*ξ*), and that when

$$\begin{array}{c} \xi =max\{k^{*}:\frac{1}{k^{*}}\sum_{k=1}^{k^{*}}\left(1-\Psi_{k}\right)\leq \delta \} \end{array}$$ (8)

where k* is the number of voxels exceeding the threshold. After determining the threshold, the result would be the final set of the significant voxels with strong evidence of effect on FA.

## Application

**Fig 1:**
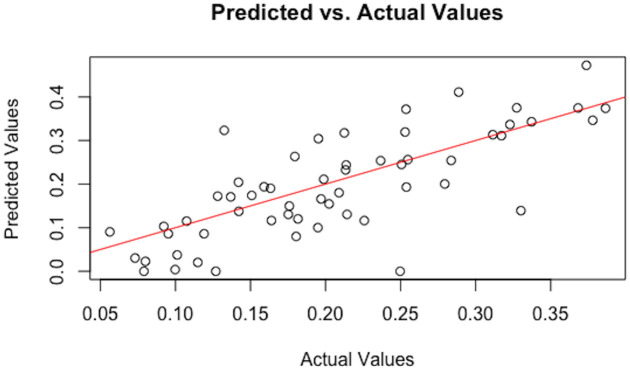
Actual FA values vs predicted values. This is a scatter plot of actual FA values vs predicted FA values for one significant voxel *v* = (150, 80, 75) across 57 individuals.

This work starts with more than 7 million voxels, but the most interesting regions are only white matter regions. By removing any black voxel and any voxel with an average of FA value lower than 0.2 in brightness which indicate that voxel has low amount of brain white matter, that leaves 601, 833 voxels of interest under 2048 possible covariate combinations of models at each voxel. By using BMS package in open-source statistical computing software R [35], we calculate PIP’s and the coefficients. The computation through the High-Performance Computing took approximately 4 days. The PIP and coefficient data was restored and the PIPs were passed through the FDR algorithm to identify significant voxels for each of the covariates. This gave 6, 646 significant voxels/covariate combinations. Then these voxels were then matched to the John Hopkins University (JHU) Atlas [36–38] map. There are 48 white matter regions of interest in brain based on the white matter JHU Atlas, which can help to determine the brain regions that present proof of changing of white matter among that significant voxels occur. Fig 1 is an example of one voxel where FA values vs predicted values. In general, it follows a linear line but this pattern is vary from voxel to voxel.

## Results

The number of significant voxels in each region for all covariate combinations as well as total number of voxels and regions with significant white matter changes are shown in S1 Table. One-way years of cocaine use (YCU) is associated with 1046 voxels significant for white matter changes, distributed across 34 JHU Brain Atlas regions; the majority of these regions are notably frontal corpus callosum, corona radiata, and internal capsule regions. One-way *GAD*1^*a*^ demonstrated a more prolific effect on the tractography data than one-way *GAD*1^*b*^ with a distinctly greater number of significant voxels (212 versus 73 total significant voxels). Nearly twice as many JHU brain regions were represented for *GAD*1^*a*^ (27) versus *GAD*1^*b*^ (14), suggesting a wider distribution of FA changes associated with *GAD*1^*a*^. Regions implicated in *GAD*1^*a*^ include primarily the splenium (19), anterior limb of the internal capsule (13), bilateral superior corona radiata (24 and 12 for right and left, respectively), superior longitudinal fasciculus (19), as well as the posterior thalamic radiation (13). Most pronounced among the *GAD*1^*b*^ clusters was the anterior limb of the internal capsule (39) with no other areas exceeding a threshold of 10 voxels.

**Fig 2:**
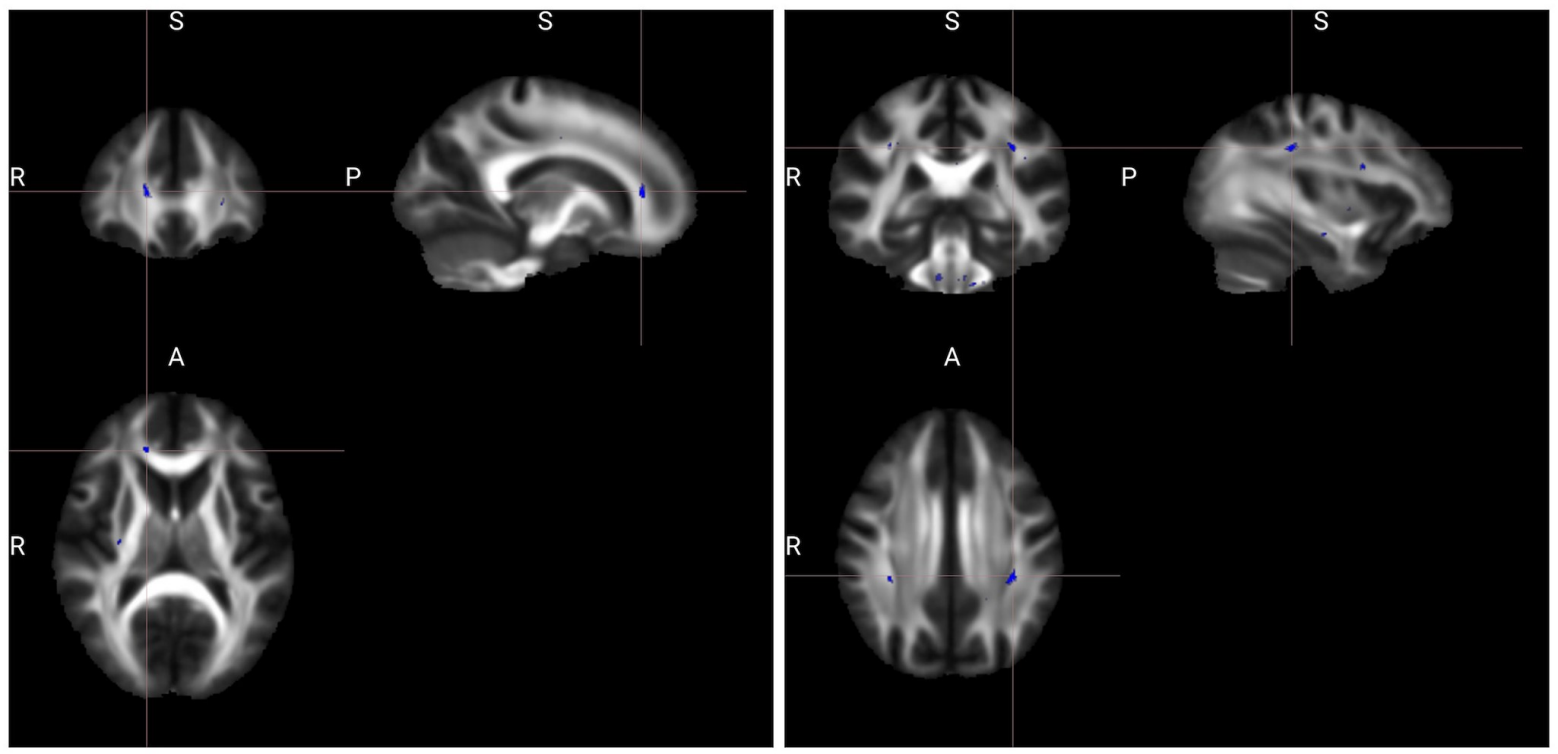
Significant regions in which the FA showing significant effect, among *GAD*1^*a*^|*YCU* (left) and *GAD*1^*b*^|*YCU* (right). The background is the average FA map. The viewer’s left side of the axial slice is the left hemisphere of the displayed brain. The clusters shown are the “thickened” version of the skeletonized results, using the FSL tbss_fill script.

With the two-way models, there was a substantial increase in significant voxels associated with *GAD*1^*b*^ versus *GAD*1^*a*^ and the number of regions with significant white matter changes was about equal. A total of 403 significant voxels for *GAD*1^*a*^|*YCU* among 33 primarily frontal and colossal JHU regions and 1553 for *GAD*1^*b*^|*YCU* among 34 mostly posterior, cerebellar, and connective tract regions. There were fourteen regions across *GAD*1^*a*^|*YCU* with more than 10 voxels, amongst which right external capsule was the largest significant region with 44 voxels. Other significant areas for *GAD*1^*a*^|*YCU* included the bilateral external capsule (44 and 33 for right and left), right anterior and right superior corona radiata (33 and 32, respectively). The body and genu of the corpus callosum (33 and 24, respectively) were also implicated. *GAD*1^*b*^|*YCU* demonstrated fifteen white matter regions with more than 10 voxels. The middle cerebellar peduncle was the largest region with 991 voxels; other cerebellar areas were implicated including right and left inferior cerebellar peduncles (49 and 62, respectively). Other major areas included in *GAD*1^*b*^|*YCU* were the right and left longitudinal fasciculus (28 and 101), right and left anterior corona radiata (26 and 13), as well as right and left medial lemniscus (44 and 51). Some genu involvement was noted as well (24), among others. In Fig 2 some significant FA findings are shown for *GAD*1^*a*^|*YCU* and *GAD*1^*b*^|*YCU*.

For the three-way model (*GAD*1^*a*^|*GAD*1^*b*^|*YCU*), there were a total of 842 significant voxels distributed across 32 regions. Eight white matter regions had more than 10 voxels in middle cerebellar peduncle (585 voxels), right anterior limb of internal capsule (24 voxels), and left retrolenticular part of internal capsule (14 voxels). Other cerebellar involvement included the right and left inferior cerebellar peduncles (38 and 51). Right and left medial lemniscus (20 and 21) as well as left superior longitudinal fasciculus (18 voxels) also were included. Perhaps most notable, the three-way model featured 14 regions with a lower number of voxels than either of the two-way models for *GAD*1^*a*^ or *GAD*1^*b*^; these regions included most of the pontine crossing tract, all corpus callosum areas, right-sided corticospinal tract, bilateral anterior corona radiata, right-sided posterior corona radiata, left-sided sagital stratum, bilateral external capsule, left-sided cingulum, left-sided stria terminalis, and right-sided superior longitudinal fasciculus. There were also 6 regions in which the three-way model had more significant voxels than either of the two-way models. These areas included fornix, right-sided anterior limb of internal capsule, left-sided posterior limb of the internal capsule, left-sided retrolenticular part of the internal capsule, right-sided cingulum, and left-sided uncinate fasciculus.

**Fig 3:**
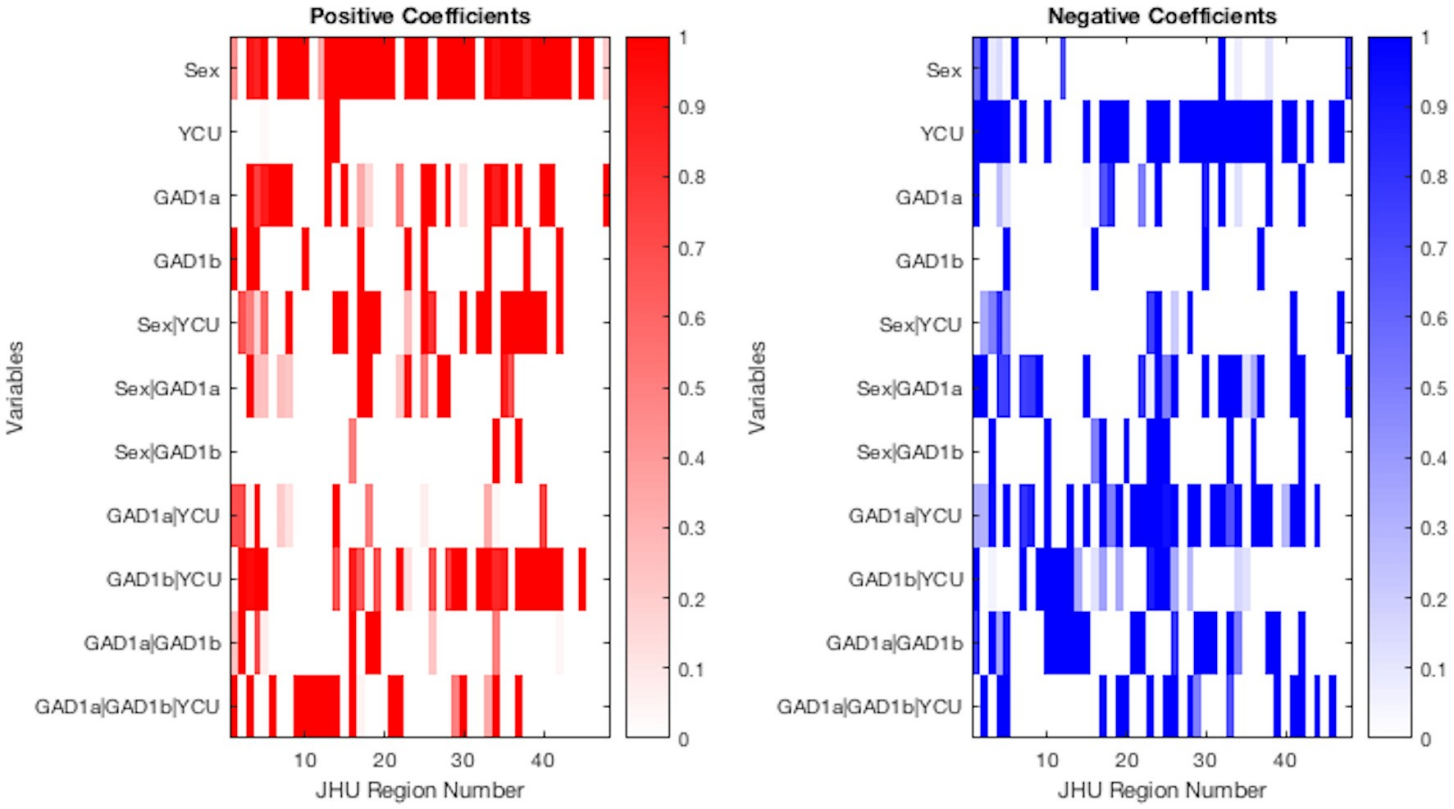
Proportion of positive coefficients (left) for significant voxels by variables across JHU Atlas regions, and negative (right). The color key represents the extent of FA alteration. White color indicates the absence of significant voxels for the specific combination of brain regions by JHU Atlas among genetic variants, Sex, and YCU.

Among the model coefficients, male sex was associated with fewer white matter changes and YCU was associated with more across essentially the entire brain as shown in Fig 3. As a one-way variable, *GAD*1^*a*^ was associated with fewer white matter changes primarily in the corpus callosum, cingulum, and superior corona radiata, demonstrated as a relative prevalence of positive coefficients. *GAD*1^*b*^ was associated with overall slightly fewer white matter changes in 10 regions throughout the brain and more white matter changes in 4 areas distributed throughout the brain.

The two-way interaction of Sex with other covariates appeared to reduce the number of regions with positive coefficients while increasing the number with negative. For example, whereas one-way *GAD*1^*a*^ on its own demonstrated more positive coefficients in the corpus callosum and frontal areas among others (23), many of these coefficients were moderated and reduced for *GAD*1^*a*^|*Sex* (11 total). For the negative coefficients, *GAD*1^*a*^ on its own featured relatively few regions with changes (12) but *GAD*1^*a*^|*Sex* featured relatively more (23).

Perhaps most notably, *GAD*1^*a*^|*YCU* demonstrated primarily negative coefficients (31) in frontal areas and connective fiber tracts including internal capsule, corona radiata, thalamic radiations, and longitudinal fasciculus; positive coefficients (14) were relatively more sparse but present primarily for cerebellum and corpus callosum. *GAD*1^*b*^|*YCU* demonstrated primarily positive coefficients (25) in corpus callosum, other thalamic radiations, and also longitudinal fasciculus; negative coefficients (18) were shown primarily for cerebellar peduncle as well as anterior and superior corona radiata. The three-way interaction *GAD*1^*a*^|*GAD*1^*b*^|*YCU* had positive coefficients (17) primarily for cerebellar peduncle and negative coefficients (17) for corpus callosum and internal capsule.

## Discussion

We applied BMA methods to DTI and genetic data collected from CUD individuals and controls, in order to investigate interaction effects of two-way and three-way variables among the *GAD*1^*a*^ and *GAD*1^*b*^ SNPs as well as clinical covariate years of cocaine use (YCU) and demographic covariate Sex in the human brain. One-way *GAD*1^*a*^ was associated with broader and more significant FA changes throughout the brain with respect to *GAD*1^*b*^, a notable 3 times difference and comparable perhaps to the roughly 4 times difference noted for the same relationship by [14]. Significant changes for one-way *GAD*1^*a*^ were more consistent with typical frontal and forebrain white matter changes associated with cocaine use disorder including corpus callosum, corona radiata, internal capsule, and thalamic projections; hindbrain white matter was shown in the cerebral peduncle, as well. One-way *GAD*1^*b*^ showed FA changes in the internal capsule alone with only scant findings elsewhere in the brain.

The inclusion of the YCU covariate revealed more substantial distribution of white matter changes for the two-way *GAD*1^*b*^|*YCU* including in the corpus callosum, additional frontal (medial lemniscus, internal capsule, corona radiata, cingulate gyrus), as well as thalamic and longitudinal fasciculus FA changes. The middle cerebellar, cerebellar peduncle FA, and superior longitudinal fasciculus changes were perhaps the most impressive with 991, combined 118 (inferior and superior cerebellar peduncle), and 129 voxels, respectively. The corpus callosal and forebrain FA changes combined with the length of use variable are perhaps more characteristic of white matter changes most typically associated with cocaine use disorder [4, 5, 7]. Increased grey matter cerebellar changes have also been associated with the length of cocaine use [8]. *GAD*1^*a*^|*YCU* was also associated with corpus callosum as well as similar frontal white matter FA changes (internal capsule, corona radiata, external capsule). Though there are some notable differences between the two models, the relative similarity of the region of FA changes between *GAD*1^*a*^|*YCU* and *GAD*1^*b*^|*YCU* suggests that YCU may be the primary driver of these FA changes. For example, *GAD*1^*b*^ on its own features only a single voxel in middle cerebellar peduncle whereas *GAD*1^*b*^|*YCU* has 991. However, there is a distinct contribution of *GAD*1^*b*^ with this particular relationship because *GAD*1^*a*^|*YCU* demonstrates only 13 significant voxels in the same region. Though there is clearly an effect of YCU on both *GAD*1^*a*^ and *GAD*1^*b*^, this particularly impressive finding would demonstrate an interaction between *GAD*1^*b*^|*YCU*.

Both two-way polymorphism and YCU models were associated with a relative increase in (Genu and Body) corpus callosum, anterior corona radiata, internal capsule, and longitudinal fasciculus involvement with respect to their one-way models. Many of these changes—particularly the corpus callosum involvement—were novel for the two-way models with respect to the one-way polymorphism models, suggesting again that YCU was the primary driver. This finding is consistent with prior research implicating corpus callosum changes associated with chronic cocaine use [7]. Notable differences include greater corticospinal tract, cerebral peduncle, and external capsule involvement for *GAD*1^*a*^|*YCU* as well as greater medial lemniscus, cerebellar, thalamic radiation, and longitudinal fasciculus involvement for *GAD*1^*b*^|*YCU*. Many of these differences were also novel for the two-way with respect to the one-way models—again, pointing to the relative contribution of the YCU variable. Again, broader frontal brain and cerebellar FA changes have been associated with cocaine use [3, 12]. White matter changes were similarly broad for *GAD*1^*a*^|*YCU* and *GAD*1^*b*^|*YCU* which featured 33 and 34 regions with white matter changes, respectively. In addition to differential involvement of certain regions, there was a substantially greater number of much larger concentration of white matter changes in the cerebellum for *GAD*1^*b*^|*YCU* and, in general, a greater posterior/mid/hindbrain-centric distribution of white matter changes. *GAD*1^*a*^|*YCU*, on the other hand, featured a larger distribution of frontal white matter changes. It is perhaps remarkable that the number of voxels with white matter changes increases by a factor of 20 for *GAD*1^*b*^ with the addition of the YCU covariate whereas the increase is only by a factor of 2 for *GAD*1^*a*^. However, if we exclude the middle cerebellar peduncle from the *GAD*1^*b*^|*YCU* model, the relative increase is only about a factor of 7. Based on the voxel-wise white matter changes data, it appears that while *GAD*1^*a*^ is associated with more white matter changes on its own in the one-way model there may be a relative protective effect associated with this polymorphism with respect to chronic cocaine use. Though there appears to be relatively fewer white matter changes on its own in the one-way, *GAD*1^*b*^ related white matter changes increase dramatically with the addition of the YCU covariate, suggesting perhaps a relative vulnerability to chronic cocaine use in individuals with the *GAD*1^*b*^ polymorphism, particularly in the cerebellum.

The model coefficients in Fig 3 provide additional evidence of the differential effects of each of the covariates. In particular, the effect of YCU on FA was negative across the majority of brain regions, as would be expected. Male sex also appears to demonstrate a protective effect against white matter changes with positive coefficients across most brain regions. The two-way interaction *GAD*1^*a*^|*YCU* featured negative coefficients for most white matter regions (31) with relatively fewer positive coefficients (10). *GAD*1^*b*^|*YCU* exhibited somewhat the opposite trend in the sense that, except for the cerebellar regions, there were relatively few negative coefficients (17) and more positive coefficients (25). The effect of the Sex covariates on FA was positive among approximately all coefficients, suggesting that male sex was a protective factor. The roughly equal number of positive (18) and negative coefficients (17) for *GAD*1^*a*^|*GAD*1^*b*^|*YCU* suggests that there isn’t a significant interaction between the polymorphisms and YCU that meaningfully increases or decreases white matter changes across the whole brain for individuals who have both polymorphisms expressed. That said, the presence of 14 regions in which the number of voxels with white matter changes were less than either two-way polymorphism model suggests that there may be a protective effect for individual regions (including corpus callosum, anterior corona radiata, among others). On the other hand, the presence of 6 regions in which the three-way model included slightly more voxels than either two-way model (including fornix and internal capsule) points to the possibility that having both polymorphisms may be associated with a deleterious effect in certain regions, as well.

For the long term goal which is to find a treatment for CUD, now we know how the polymorphisms interact with each other and with lifetime cocaine use, also we know which is more effective than others. For future work, This can provide genetic testing that allowance position to better prescribe a treatment. Also allowance the doctors really understanding what is in the patient’s future given their genetic profile, which can be used at least as personalized medicine.

## Conclusion

Bayesian analysis was used to analyze the relationships between genetic data, sex, years of cocaine use, and fractional anisotropy white matter changes. The underlying advantage of this modeling approach is taking into account the model uncertainty through Bayesian model averaging. This method was building on genetic data from the previous work of [14] exploring in greater detail the relative effects of years of cocaine use as well as the possession of *GAD*1^*a*^ and *GAD*1^*b*^ polymorphisms. The primary novel addition in this study is the added consideration of years of cocaine use and sex in a series of two- and three-way models of the interaction of the genetic variants on brain white matter in users of cocaine. Specifically, the goal of this work was to identify brain regions that present strong evidence for differential diffusion patterns across the two-way and three-way interactions of *GAD*1^*a*^|*YCU*, *GAD*1^*b*^|*YCU*, and *GAD*1^*a*^|*GAD*1^*b*^|*YCU*. Ancillary goals included evaluating the effect of sex on white matter changes; in these data, male sex appears to have a mild protective effect. *GAD*1^*a*^ appears to be associated primarily with frontal white matter changes with only a moderate increase in voxel-wise volume with chronic cocaine use. *GAD*1^*b*^, on the other hand, was associated with a more dramatic increase in cerebellar white matter changes as well as other primarily mid/hindbrain regions based on the voxel-wise data. The presence of 14 regions in the three-way model which feature lower voxel counts than either of the two-way polymorphism models points to a possible protective effect of having both polymorphisms. The model coefficients indicate that *GAD*1^*a*^|*YCU* is associated with more negative white matter changes and *GAD*1^*b*^|*YCU* with more positive. The three-way model *GAD*1^*a*^|*GAD*1^*b*^|*YCU* demonstrated fewer negative coefficients than either two-way model as well as a cluster of novel positive coefficients in the medial lemniscus and cerebellar peduncle not shared by the two-way models—again, pointing to a possibly protective effect in having both polymorphisms.

However, since the values of the response variable *y*(*v*) are belong to the standard unit interval (0, 1), then the linear regression is not the most appropriate method to analyze the data. That because the predicted values could be out of the interval (0, 1) when using linear regression. One of the suggestions solution of this issue is to transform the response variable to be values on the real line, then analyze the transformed data using linear regression. However, this method has some weaknesses such as: the regression parameters are explicated in terms of the transformed data and it is difficult to interpret it in terms of the un-transformed data. Moreover, the distributions of proportions are not symmetric, then conclusions relying on the normality assumption could be invalid. In order to overcome this problem, a regression model for continuous variables that belong to the standard unit interval (0, 1) has been proposed by [39]. This proposed model is under an assumption that the response variable is Beta distributed, and the model is called Beta regression model. The underlying advantage to use the Beta regression model is the flexibility that achieved by the assumption of the response variable which is following Beta distribution.

## Supporting information

S1 Table Number of voxels with white matter (FA) changes by JHU Brain Atlas region for the variables and covariates. (XLSX)
